# Granulomatosis with Polyangiitis with Myocarditis and Ventricular Tachycardia

**DOI:** 10.1155/2017/6501738

**Published:** 2017-08-20

**Authors:** Ramy Magdy Hanna, Eduardo Lopez, James Wilson

**Affiliations:** ^1^Division of Nephrology, Department of Medicine, David Geffen School of Medicine, Los Angeles, CA, USA; ^2^Division of Nephrology, Department of Medicine, Kaiser Permanente Medical Center, Panorama City, Los Angeles, CA, USA; ^3^Surgical Consultative Nephrology, UCLA Stone Center, Los Angeles, CA, USA; ^4^David Geffen School of Medicine, Los Angeles, CA, USA

## Abstract

Granulomatosis with polyangiitis (GPA), previously known as Wegener's granulomatosis, is a pulmonary-renal syndrome affecting small and medium sized blood vessels. The disease has a prevalence in studies ranging from 3 to 15.7 cases per 100,000, with a noted increasing incidence and prevalence in more recent studies. Pulmonary manifestations include hemorrhage, lung cavitary lesions, and pulmonary fibrosis. Within the kidney, GPA is known to cause rapidly progressive pauci-immune crescentic glomerulonephritis. Rare and severe cardiovascular manifestations include pericarditis, arrhythmias, myocarditis, and aortic valve disease. Our patient is a 43-year-old female with typical pulmonary and renal lesions from GPA and also acute myocarditis, multiple episodes of ventricular tachycardia, and a severe reactive thrombocytosis.

## 1. Introduction

Granulomatosis with polyangiitis (GPA) is an autoimmune inflammatory disorder affecting the small and medium sized blood vessels of the body [1]. It is part of a larger group of antineutrophil cytoplasmic antibody (ANCA) associated autoimmune vasculitic syndromes including Eosinophilic Granulomatosis with Polyangiitis (EGPA), polyarteritis nodosa, and microscopic polyangiitis [2]. Typically, this disorder is associated with a positive antineutrophil cytoplasmic antibody (c-ANCA) but this is not always the case [2–4]. The incidence of the disease is noted to be rising and the prevalence previously evaluated at 3 cases per 100,000 has now been noted to be as high as 15.7 cases/100,000 patients [1, 5].

There is a wide spectrum of well known pulmonary manifestations including cavitary changes in the lungs that can mimic infectious diseases such as tuberculosis [6] and pulmonary hemorrhage. Pulmonary fibrosis with loss of lung function can occur in chronic cases of GPA [7]. Sinus involvement is another commonly seen manifestation associated with GPA [8]. The renal manifestations are typically a rapidly progressive crescentic necrotizing glomerulonephritis that is caused by small vessel vasculitis within glomeruli [1]. The c-ANCA antibody, specifically the antiproteinase-3 antibody, is thought to have a pathological role in the development of the inflammation in the small and medium sized arteries of the body. The American College of Rheumatology (ACR) classification criteria include the following: (1) abnormal urinary sediment (red cell casts or greater than 5 red blood cells per high power field), (2) abnormal findings on chest radiograph (nodules, cavities, or fixed infiltrates), (3) oral ulcers or nasal discharge, and (4) granulomatous inflammation on biopsy. The presence of 2 or more of these 4 criteria was associated with a sensitivity of 88.2% and a specificity of 92.0% [9].

GPA is not limited to the pulmonary or renal systems; the inflammation can strike in the medium sized arteries of any organ in the body. There are even rare case reports of large vessel vasculitis such as cases of renal artery stenosis [1]. It is also not surprising that there have been many case reports of organ involvement in the tympanic membranes, which are contiguous with the sinuses [10]. Facial nerve palsy and diverse neurological manifestations have also been reported [10, 11]. GPA has also reported to cause endocrine gland pathology secondary to the vasculitis, and there are case reports of adrenal infarction in the setting of active disease [12, 13].

Cardiac manifestations such as pericarditis due to serositis have been described in GPA previously [14–19]. Grant et al. detailed three different cases of cardiovascular complications related to inflammation secondary to c-ANCA positive vasculitis that required cardiothoracic surgical intervention (two with pericarditis, one with tamponade) [20]. Cardiomyopathy with depressed ejection fraction (EF) has also been infrequently reported in the literature [21]. Ventricular arrhythmias triggered by active vasculitis are very uncommonly observed and very rarely reported. Conduction abnormalities, valvular disorders [20, 22], unstable angina secondary to vasculitis, and subclinical myocarditis [23] are among the uncommon cardiac manifestations of GPA.

This case illustrates simultaneously in one patient several of the rarer cardiac manifestations seen in GPA such as depressed ejection fraction, myocarditis, and life threatening ventricular arrhythmias.

## 2. Case Presentation

Our patient is a 43-year-old female without known medical history who presented to Olive-view UCLA medical center with a chief complaint of shortness of breath. She was initially diagnosed with asthma when her complaints were evaluated and was treated with steroid courses without effect. She then developed multiple bouts of otitis media and sinusitis and was given multiple courses of antibiotics with only partial relief of her symptoms. She was diagnosed with chronic mastoiditis at an outside hospital given her chronic infections and underwent a mastoidectomy at an outside facility.

**Figure 1:**
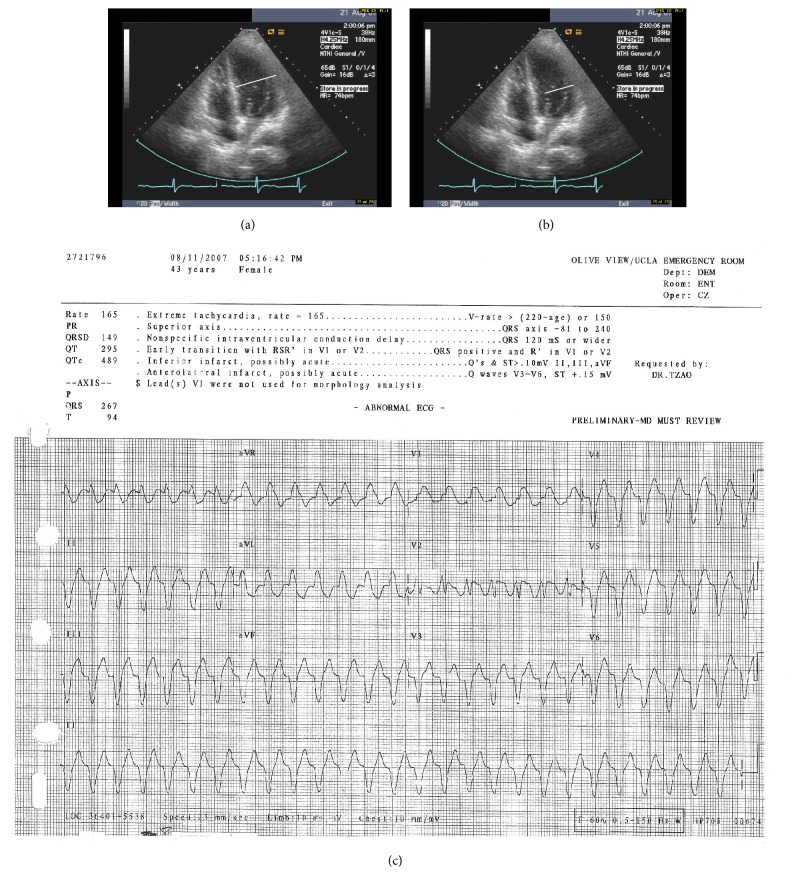
(a) Patient 08/11/2007 four-chamber view echocardiogram showing a dilated myocardium and an ejection fraction of 25% note sinus rhythm; (b) Patient 09/04/2007 four-chamber view echocardiogram showing less dilatation, movement of myocardium inward during systole, and an ejection fraction of 55%; (c) EKG showing monomorphic ventricular tachycardia due to GPA myocarditis and hyperkalemia.

Her symptoms continued and she developed chest pain and shortness of breath and noted decreasing urine output. Upon presentation to the emergency department, she developed a wide complex tachycardia consistent with monomorphic ventricular tachycardia (see Figure 1). The patient became apneic and was intubated, cardioverted several times, and given multiple doses of amiodarone to stabilize her arrhythmia. A venous blood gas showed a potassium of 6.7 mg/dL [normal range = 3.7–5.2 meq/L] and troponins drawn before cardioversion were initially positive at 2.36 mg/dL [normal range < 0.01 ng/ml].

Customary treatment for hyperkalemia (intravenous insulin and Dextrose 50%, oral Kayexalate, and intravenous calcium gluconate) was administered. The troponin increased to 33.6 ng/ml and an echo was done and showed global hypokinesis with a greatly depressed EF of 25% [normal range = 55–65%]. It is important to note that the initial echo was conducted while patient was in sinus rhythm not during an arrhythmia. Labs from day of admission showed global elevation of inflammatory markers with a high sensitivity C-reactive protein (Hs-CRP) that was extremely elevated at 383.3 mg/L [normal range = 1–3 mg/dL] and an Erythrocyte Sedimentation Rate (ESR) that was also very elevated at 130 mm/hour [normal range = 0–20 mm/hour]. Urinalysis showed a pH of 7 [normal range = 6.5–8], specific gravity of 1.014 [normal range = 1.003–1.035], 2+ protein [normal range = 0 to trace], 2+ glucose in a nondiabetic [normal range = 0], negative ketones [normal range = negative], negative bilirubin [normal range = negative], 0.2 urobilinogen [normal range = 0–8 mg/dL], large blood [normal range = negative], negative nitrites [normal range = negative], negative leukocyte esterase [normal range = negative], 3 white blood cells/high power field (hpf) [normal range = 0–10 wbc/hpf], 343 red blood cells/hpf [normal range = 0–4 rbc/hpf], 4 squamous epithelial cells/hpf [normal range = 2–5 cells/hpf], and few amorphous crystals/hpf [normal range = negative].

**Figure 2:**
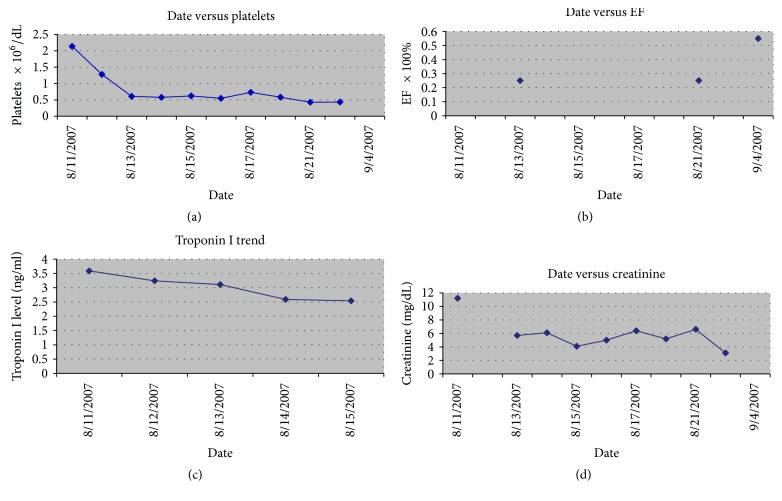
(a) Graph of trend of platelet count versus time for initial hospitalization for patient. (b) Graph of trend of ejection fraction (EF) versus time for initial hospitalization for patient. (c) Graph of downtrend of troponin after peak value of 33.6 ng/ml versus time for initial hospitalization for patient. (d) Graph of trend of serum creatinine versus time for initial hospitalization for patient.

The patient was transferred to intensive care and emergent dialysis was performed due to hyperkalemia and uremia (BUN = 125 [normal range = 7–20 mg/dL] and Cr = 11.6 [normal range = 0.7–1.3 mg/dL]) and metabolic acidosis. The patient's platelets were noted to be profoundly elevated and were noted on presentation to be as high as 2.136 million/dL [normal range = 150,000–450,000] and decreased to 1.3 million/dL by day 2. Plasmapheresis was performed on hospital day two, and the patient was treated with hydroxyurea 1500 mg daily. For trends in the labs and echocardiogram data pertinent to this presentation please see Figures 1 and 2 and Table 1.

The patient improved afterwards and was extubated four days later. A cardiac catheterization showed no significant obstructive coronary artery disease. She was treated for GPA with pulse dose steroids (hydrocortisone) and intravenous cyclophosphamide at 750 mg. Her ejection fraction continued to slowly improve until it increased to 50% after 30 days. It was decided that the patient likely had GPA myocarditis or coronary vasculitis given initially positive troponins, and this has been previously reported in the literature [21]. Her platelets also continued to decline until they approached the upper bound of normal after three weeks of treatment.

A bone marrow biopsy done six days after admission showed some clusters of atypical megakaryocytes that could be consistent with essential thrombocytosis; thus the patient was started on plateletpheresis and hydroxyurea. The Jak-2 kinase study which is 50% sensitive in essential thrombocytosis (ET) was negative. The patient's bone marrow was negative for any karyotypic abnormality or for the BCR-Abl mutations. There was no evidence of plasma cell changes or other leukemoid changes. The thrombocytosis was deemed to be reactive due to vasculitis.

The patient's sinus passages were also biopsied and the pathology report showed granulomatous inflammation and histiocytes consistent with GPA. The patient's renal status was stabilized and she continued to receive regular hemodialysis to control her electrolytes and fluid balance. A kidney biopsy was obtained and was consistent with pauci-immune glomerulonephritis with 85 to 90% of glomeruli showing crescentic changes. This is consistent with a rapidly progressive glomerulonephritis of the kidney that is typical of GPA [1]. Our patient also met three of the four clinical criteria for GPA [9] as well as having a positive c-ANCA proteinase-3 antibody titer of 1 : 100 [normal range < 1 : 20 titer]. She received two doses of cyclophosphamide and required subsequent treatment with rituximab with complete remission of her symptoms for the last four years (see Figure 2 and Table 1).

## 3. Discussion

This case illustrates typical and atypical signs of GPA. The patient in question presented with acute renal failure caused by pauci-immune crescentic glomerulonephritis. Her ENT manifestations were typical of GPA; the patient's respiratory symptoms were less typical. The unusual cardiac manifestations of GPA discussed here are noteworthy especially the myocarditis and the life threatening ventricular tachycardia observed. While this patient did not have Positron Emission Tomography (PET) at the time of presentation, these scans are now being used to evaluate the extent of myocarditis in GPA [19]. There are also reported cases of rituximab use to ameliorate the myocarditis in patients with GPA pericarditis, myocarditis, and related cardiac conduction system disease [23]. The thrombocytosis was severe in its magnitude but not unexpected and previously observed in the literature. The resolution of this thrombocytosis with appropriate treatment for vasculitis helps confirm this clinical impression.

Several points made us consider that the patient actually had myocarditis. The first point was the drop in EF to 25. The second was the troponin level being quite elevated at 2.63 ng/mL prior to cardioversion without any evidence of a significant blockage being found on angiography. While demand ischemia may have been present to some degree during the ventricular tachycardia, the decreased ejection fraction is not accounted for by this explanation. While it could be argued that some of the subsequent troponin elevation was due to cardioversion, the magnitude of troponin elevations in the literature noted after cardioversion is generally mild on order of 0.02 to 0.04 ng/ml [24]. The increase of the troponin to 33.6 ng/mL is not explained by either demand ischemia or cardioversion, with such a large elevation tending to suggest involvement of a significant proportion of the myocardium. These observations make the diagnosis of myocarditis reasonable on a clinical basis. The improvement of the ejection fraction with treatment for GPA also offers some circumstantial evidence for cardiac inflammation that improved with immunosuppressive therapy.

Unfortunately, PET studies were not available in our facility at that time to definitively diagnose this event and an endomyocardial biopsy would have been too hazardous given how ill the patient was. The hyperkalemia that developed due to acute kidney injury may have contributed to or alternatively caused the ventricular arrhythmia as well. Given the above observations about the elevated troponin markers and decreased EF in the absence of any coronary obstruction, we cautiously conclude the patient had some measure of cardiac inflammation and cannot rule out that hyperkalemia played a role in this presentation as well.

According to McGeoch et al., 3.3% of patients with GPA had myocardial involvement [25], so while it remains rare it is not an unprecedented condition. Some cases may simply present with mild chest pain or shortness of breath as was noted in Munch et al. [26]. The recognized rates of myocardial involvement in GPA may increase further if asymptomatic patients are screened who may be having silent cardiac involvement in this systemic vasculitis [25].

The patient is currently being seen at our medical center, she was initially maintained on mycophenolate mofetil (Cellcept) and was later switched to methotrexate. Her renal function has stabilized and she has not required hemodialysis, as her serum creatinine is 0.87, and she has a normal ejection fraction of 55% (see Table 1). A slow withdrawal of immunosuppression is being implemented at this time. Recent data did show it to be safe to slowly withdraw immunosuppression in cases of GPA in remission after 15 to 18 months of active therapy [27].

## Figures and Tables

**Table 1:** Table of trends and clinical timeline of events, following trends from 2007 to 2011.

Lab trends seen in case of multisystem GPA					
Date	EF*∗*100%	Cr mg/dL	Plts (×10^6^)	Peak troponin I	Event

8/11/2007		11.2	2.136	33.6	Initial arrival (1st troponin 2.63 ng/ml before Vtach and cardioversion), peak after cardioversion was 33.6 ng/ml given hydrocortisone 100 mg iv TID (Stress dose), hydroxyurea 1500 mg, and HD #1

8/12/2007			1.278	3.24	Given plasmapheresis session #1 and HD #2, started on prednisone 60 mg

8/13/2007	0.25	5.7	0.608	3.11	Second echo checked

8/14/2007		6.1	0.577	2.59	

8/15/2007		4.1	0.619	2.54	Last troponin checked at first hospitalization

8/16/2007		5	0.545		

8/17/2007		6.4	0.728		

8/18/2007		5.2	0.58		First cyclophosphamide dose given 750 mg

8/21/2007	0.25	6.6	0.428		Third echo checked

8/24/2007		3.1	0.432		

9/4/2007	0.55				

9/18/2007		1.6	0.351		

8/12/2008	0.55	1.42	0.367		Early 2008 second cyclophosphamide course, switched to IV Rituxan and then Cellcept

1/20/2011	0.55	0.87	0.4		Small pericardial effusion seen on Echo, switching to maintenance methotrexate

Cr = serum creatinine, EF = ejection fraction, HD = hemodialysis, IV = intravenous, and Plts = platelets.
